# Vancomycin-Resistant Enterococci and Extended-Spectrum β-Lactamase-Producing Bacterial Colonization of the Cervix after Emergency Cerclage: Is It Safe?

**DOI:** 10.3390/antibiotics10080933

**Published:** 2021-07-31

**Authors:** Won-Kyu Jang, Jin-Gon Bae

**Affiliations:** Department of Obstetrics and Gynecology, Keimyung University School of Medicine, Dongsan Medical Center, Daegu 42601, Korea; cindeln@naver.com

**Keywords:** vancomycin-resistant enterococci, VRE, extended-spectrum beta-lactamase, cerclage, cervical, cervix incompetence

## Abstract

Antimicrobial resistance is currently becoming a global threat to human health. We performed a retrospective study on patients who underwent emergency cerclage between January 2016 and December 2018 at the Dongsan Medical Center. Cervical culture was first performed before surgery to confirm that there was no infection and was repeated on days 1, 4, and 7 after surgery. A total of 85 pregnant women underwent emergency cerclage. Among them, six patients had vancomycin-resistant enterococci (VRE) colonization in the cervix after cerclage, and 23 patients developed extended-spectrum β-lactamase (ESBL)-producing bacterial colonization in the cervix. The average gestational age at delivery was lower in the VRE group. Neonatal death was also significantly higher in the VRE group. The rate of occurrence of early-onset sepsis was also higher in the VRE group, and both VRE and ESBL-producing bacterial colonization cases in which early-onset sepsis occurred resulted in neonatal death. The prognosis of cervical VRE colonization after cervical surgery was poor, whereas the prognosis of ESBL-producing bacterial colonization in the cervix did not differ significantly from that of the control group. However, careful neonatal treatment is required considering that early-onset sepsis is fatal to the newborn.

## 1. Introduction

Preterm birth is highly associated with perinatal morbidity [1]. Cervical insufficiency is a condition in which the cervix dilates or when the amniotic membrane protrudes out of the cervix in the absence of uterine contractions during the midtrimester of pregnancy [2,3]. Cervical insufficiency is a risk factor for miscarriage or premature birth in the second trimester [4]. Cervical cerclage is a surgical procedure performed for pregnant women who currently have or have had cervical insufficiency, and it is performed as a very effective and general treatment to prevent preterm birth [5,6].

Antimicrobial resistance (AMR) is currently becoming a global threat to human health [7]. *Enterococcus* species is a component of the human gut flora and is found naturally in the birth canal of women. Vancomycin-resistant enterococci (VRE) are more resistant to treatment than their susceptible counterparts. In addition to the development of enterococci with high levels of resistance to penicillin and aminoglycosides, the emergence of vancomycin resistance in enterococci poses serious challenges and difficulties for physicians treating patients infected with these microbes [8]. Extended-spectrum β-lactamase (ESBL) is an enzyme that changes and has the ability to inactivate beta-lactam-containing antibiotics. Bacteria that produce ESBL are resistant to certain types of antibiotics, such as penicillin, cephalosporin, and monobactam. Since blaESBLs are located in the plasmid, it can be transmitted from one bacterial species to another or from strain to strain. Therefore, prevention and treatment of these ESBL-producing bacteria are regarded as an increasingly important task [9,10,11].

Very few articles have been published on infections due to VRE and ESBL-producing bacteria during pregnancy. Among them, one of the few studies that reported on vaginal colonization of VRE during pregnancy was that of Miller et al [12]. In addition, one of the few studies that reported on vaginal colonization of ESBL-producing bacteria during pregnancy was that reported by Foessleitner et al [13]. Thus, while there are a few examples of papers that have reported on AMR during pregnancy, there have been no reports on the occurrence of VRE or ESBL-producing bacteria in the group that underwent cerclage during pregnancy and the clinical features and prognosis of these infections in these groups.

Therefore, we report cases of cervical colonization of VRE and ESBL-producing bacteria that occurred in the group of women who underwent cerclage in our hospital, and we review the clinical manifestations and prognosis of these infections for the first time.

## 2. Results

A total of 85 pregnant women underwent emergency cerclage. Among them, 6 patients had VRE colonization in the cervix after cerclage, and 23 patients developed ESBL-producing bacterial colonization in the cervix. There was no significant difference in basic characteristics between the variables in these groups except for body weight and body mass index (Table 1).

In the VRE colonization group, the species identified in cervical cultures were all *Enterococcus faecium*. In the ESBL-producing bacterial colonization group, *Escherichia coli* was identified in 19 cases (82.6%), and *Klebsiella pneumoniae* was identified in four cases (17.4%) in cervical cultures. In the VRE colonization group, all cultures were first identified 7 days after cerclage. In the ESBL-producing bacterial colonization group, 12 cases (52.2%) were identified for the first time in the culture performed 4 days after cerclage, while the others were identified 7 days after cerclage. The number of cases in which the amniotic membrane was exposed to the vagina before cerclage was significantly higher in the VRE colonization group. The total cervical length measured after cerclage was not significantly different between the three groups, but the length from the cervical stitch to the most external part of the cervix was significantly shorter in the VRE colonization group (Table 2).

Although cerclage was already performed, a repeat cerclage was performed when the amniotic membrane was exposed to the vagina again due to weakened or torn stitches. These cases were observed more in the VRE colonization group. The gestational age at delivery and the interval from surgery to delivery also differed significantly between the three groups. The average gestational age at delivery was lower in the VRE group, and the interval from surgery to delivery was shorter; however, there was no significant difference between the ESBL-producing bacteria group and the control group. As such, there was a significant difference in the birth weight. The neonatal mortality rate was also significantly higher in the VRE group. The rate of occurrence of early-onset sepsis in the neonate was also higher in the VRE group, and both VRE and ESBL-producing bacteria cases where early-onset sepsis in neonate resulted in the neonatal death (Table 3). In three cases of neonatal death, all species found colonizing the maternal vagina were also found in blood cultures of the neonates. In particular, in the case of a neonate with maternal ESBL-producing colonization, the same bacteria were also identified in the neonate’s skin, gastric juice, and bronchial fluid.

## 3. Discussion

The present study aimed to investigate the effect of cervical colonization by VRE or ESBL-producing bacteria after cerclage on the clinical features of pregnancy and neonatal outcomes. Cervical colonization by VRE occurred more frequently when rescue cerclage was performed, the probability of undergoing repeat cerclage was higher, and the neonatal outcome was also poor due to the high probability of giving birth at an earlier gestational age. On the other hand, ESBL-producing bacterial colonization did not differ significantly in clinical features and neonatal outcome compared to the control group, but the prognosis was poor if early-onset sepsis occurred in the newborns.

Enterococci species are part of the normal flora and are commonly encountered in the gastrointestinal tract [14]. Multidrug resistance has emerged in this important pathogen due to the increased use of broad-spectrum antibiotics used in hospitals and the widespread use of invasive devices [15,16]. As they usually have widespread resistance to broad-spectrum antibiotics, these antibiotic-resistant enterococci are the main cause of hospital-acquired infections in the blood and urinary tract [17], among which VRE is clinically highly associated with high mortality [17,18]. The only available report of such dangerous VRE during pregnancy in the literature was a paper published by Miller et al., and this paper reported that the rate of VRE found by chance during prenatal screening was 3.8%, although they did not present results for pregnancy outcome and neonatal outcome [12]. Similarly, this is the first paper to report on VRE that occurred after cerclage during pregnancy. In our paper, the colonization of VRE in the cervix does not appear to be accidental colonization. Although it was not detected before cerclage, it is considered to be a hospital-acquired infection from the pattern found later. In spite of continued standard in-hospital disinfection and management for infection control such as VRE, approximately six cases occurred during the study period. As our results show, in the group with VRE colonization of the cervix that occurred after cerclage, there was not only the probability of performing repeat cerclage but also significantly worse pregnancy outcomes and neonatal outcomes. It was initially thought that VRE colonization occurs more easily when rescue cerclage is performed. Rescue cerclage is performed when the amniotic membrane is exposed to the vagina, and it is necessary to push the amniotic membrane back into the cervix when closing the cervix with a thread. Amnioreduction is a commonly used method to reduce the amniotic fluid pressure so that the amniotic membrane can rise well. This is used together with the method of pushing back the amniotic membrane into the uterus through several devices [19]. The use of these procedures and devices is not performed in general cerclage, which is thought to be the cause of the increase in the incidence of VRE. Looking at past studies that report the use of an invasive device increases the incidence of VRE [18], this is likely to be one of the causes of VRE colonization in the current study.

Published papers on ESBL-producing bacterial colonization during pregnancy are very rare, and the effect of the colonization of ESBL-producing bacteria on pregnancy is unknown. Foessleitner et al. reported that vaginal colonization of ESBL-producing bacteria during pregnancy appears to be associated with preterm birth and preterm premature rupture of membranes (pPROM) and is an important risk factor for neonatal transmission [13]. To the best of our knowledge, this paper is the first study on ESBL-producing bacteria in pregnant women who underwent cerclage, and it showed different results from those published by Foessleitner et al. There were no statistically significant differences in pPROM and preterm birth rate between the ESBL-producing bacteria group and the control group, neither were there any significant differences in other pregnancy outcomes and neonatal outcomes between the two groups. This is clearly different from the ESBL-producing bacteria identified during screening in the previous paper in that we identified a newly colonizing ESBL-producing bacterial strain after the cerclage was performed, and this is presumably because the mechanism of occurrence of ESBL-producing bacteria is different. Pessoa-Silva et al. reported that third-generation cephalosporin and aminoglycoside combination therapy is an independent risk factor for the development of ESBL-producing bacteria [20], and Crivaro et al. also reported that ampicillin/gentamicin combination therapy in neonates is associated with the occurrence of ESBL-producing bacteria [21]. Judging from the reports on the occurrence of ESBL-producing bacteria due to the use of these antibiotics, we used a combination of a third-generation cephalosporin, clarithromycin, and metronidazole for a week after cerclage was performed; this may have played a major role in the development of ESBL-producing bacteria. Although the exact reason is not known, it is thought that the differences between these causes may show the difference in pregnancy outcome and neonatal outcome when compared with previous studies.

However, it should be pointed out in this paper that the early-onset sepsis that occurred in the group with colonization of VRE and ESBL-producing bacteria was fatal. In the VRE colonization group, although the incidence of early-onset sepsis was 33.3% and that of ESBL-producing bacteria was only 4.8%, neonatal death occurred in all cases (100%) of early-onset sepsis. Of course, there may be bias in interpretation due to the small number of cases, but neonatal death was not reported in early-onset sepsis caused by the vaginal colonization of ESBL-producing bacteria reported by Foessleitner et al [13]. Furthermore, to our knowledge, there is no existing report on early-onset sepsis caused by VRE colonization, so it is difficult to make comparisons with our paper; the prognosis of early-onset neonatal sepsis generated from VRE and ESBL-producing bacterial colonization of the cervix after cerclage is considered to be poor. If VRE and ESBL-producing bacterial colonization of the cervix is found after cerclage, it appears that parents need sufficient explanations and warnings about this. In addition, it appears necessary to receive strict monitoring and care in the neonatal intensive care unit immediately after birth, and efforts should be made to improve neonatal outcomes through the selection of appropriate empirical antibiotics.

Intra-amniotic infection confirmed by amniocentesis has been reported in up to 52% of pregnant women with cervical insufficiency [22,23,24,25,26]. These pregnant women show symptoms, such as chorioamnionitis, premature membrane rupture, and vaginal bleeding that may require delivery after cervical cerclage, which are associated with poor pregnancy outcomes [22]. A study showed that the administration of indomethacin and antibiotics after surgery in midpregnant women who underwent cervical cerclage can extend the gestation period by 28 days [27]. Therefore, most obstetricians use antibiotics after performing cervical cerclage. A recently published study by Oh et al. also reported that the use of ceftriaxone, clarithromycin, and metronidazole resolved the intra-amniotic infection and inflammation by 75% in pregnant women with cervical insufficiency [28]. Based on the findings of these, antibiotics were used after surgery in our study. Although the negative effects of antibiotics, such as the occurrence of multidrug-resistant bacteria, are well-known [29,30], preterm birth due to infection after cerclage is an important issue directly related to the survival of neonates, and the use of antibiotics is considered essential. In general, it is well-known that a fetus must be delivered after 24 weeks of gestation for viability [31] because preterm birth increases perinatal morbidity [32]. In cervical cerclage, which is mainly performed before 24 weeks of gestation, preterm birth due to infection caused by not using antibiotics is very likely to be fatal for the neonates. As such, the neonatal outcome due to preterm birth is a very important part of predicting the prognosis in the obstetric field. To prevent preterm birth, cerclage operation is performed on pregnant women with cervical insufficiency, and the use of antibiotics after surgery must also be performed. It is important to consider that cervical culture after cerclage must be performed, as shown in the findings of multidrug-resistant bacteria in this study. If multidrug-resistant bacteria are found in cervical culture, a further cerclage may be performed, or the birth weight of the fetus may decrease due to preterm birth. In particular, if there is a cervical colonization of multidrug-resistant bacteria, this can be transmitted to neonates during delivery [33], as shown in the results of this study. Early-onset sepsis in neonates is extremely fatal and can lead to neonatal death [34,35].

The limitations of this paper include the fact that its retrospective design reduces its statistical power, that the study was conducted through the review of medical records, and that the number of groups of VRE colonization is small. However, we think that the strength of the collection of six rare cases and the peculiarities of the pregnant women who underwent cerclage is rather strong.

## 4. Materials and Methods

Our institutional review board approved the protocol for the present study. We retrospectively analyzed the data of a patient cohort of 85 women who underwent emergency cerclage from January 2015 to December 2018 at the Dongsan Medical Center, Daegu, Korea. In the guidelines published by the American College of Obstetricians and Gynecologists or the Royal College of Obstetricians and Gynecologists [2,3], emergency cerclage was defined such that it included both ultrasound-indicated cerclage (UIC) and rescue cerclage. UIC was performed on pregnant women with a history of preterm birth due to preterm labor at <34 weeks when the patient’s cervical length was ≤25 mm at 14–24 weeks of gestation. Rescue cerclage was performed when the fetal membrane was exposed to the vagina during physical examination using a speculum before 24 complete weeks of gestation [3]. Cervix length was measured using a transvaginal ultrasound (Samsung Medison WS80A). We excluded patients with premature rupture of membranes, chorioamnionitis, multiple pregnancies, fetal anomalies, and chromosomal abnormalities. Cerclage was performed under spinal anesthesia using the Mersilene tape. Cervical cerclage is a well-known surgical procedure that is used to approach the vagina and stitch the neck area of the cervix in turns [36]. After surgery, antibiotics were used to prevent intra-amniotic inflammation and infection, and prophylactic antibiotics (third-generation cephalosporin, clarithromycin, and metronidazole) were used for one week, as in the study of Oh et al [28]. If the cervical stitch was torn or opened after the surgery due to preterm labor or other causes, and the fetal membrane was exposed to the vagina, cerclage operation was performed again to improve the neonatal outcome.

If there is an infection around the cervix, intra-amniotic inflammation and infection may happen, which may worsen the neonatal outcome due to preterm birth [28]. The timing of cervical cultures before and after cerclage is different for each hospital [37,38,39], but in this study, following our hospital’s protocol, cervical culture was first performed before cerclage to confirm no growth of bacteria and again at 1, 4, and 7 days after cerclage to confirm the growth of bacteria. Cervical culture was performed using swab samples of cervical mucus and cells through vaginal examination. Bacterial culture of the cervix was performed using a blood agar plate, and the identification of colonies grown on the blood agar plate was carried out using MALDI-TOF VITEK^®^MS (bioMérieux, Marcy l’Étoile, France). Antimicrobial susceptibility test was analyzed by Vitek 2 AST for gram-positive bacteria and BD Phoenix automated susceptibility system (BD Diagnostic Systems, Sparks, MD) for gram-negative bacteria. The BD Phoenix ESBL test uses growth response to select expanded-spectrum (cefpodoxime) and broad-spectrum (ceftazidime, ceftriaxone, and cefotaxime) cephalosporins, with or without clavulanic acid, to detect the production of ESBL. Resistance was defined according to the criteria of the Clinical and Laboratory Standards Institute (CLSI) [40].

Bacterial colonization was defined as the presence of organisms without symptoms and no tissue damage or invasion [29]. In a cervical culture performed after surgery, if there are bacteria commonly found colonizing the female genital tract with no specific clinical infectious signs or symptoms, they are classified as colonization. Among several species that can cause vaginal colonization during pregnancy [41], we analyzed only *Enterococcus* spp., *E. coli*, and *Klebsiella pneumoniae* in this study.

Medical record data were reviewed retrospectively. The patients were divided into three groups: the first group was that in which VRE was identified, the second was that in which the ESBL-producing bacteria were identified, and the third group was classified as a case with no bacterial growth, and it was defined as a control group. We also collected data on patients’ obstetric history, cervical length at the time of surgery, cervical length 1 day after surgery (i.e., total length, length below knot), obstetrical complications, and neonatal outcomes.

Statistical analyses were performed using the Statistical Package for the Social Sciences (SPSS 25.0; IBM Corporation, Armonk, NY, USA). Continuous variables were expressed as mean ± standard deviation, as well as descriptive statistics. The one-way analysis of variance, Kruskal–Wallis test, Mann–Whitney U test, Student’s t-test, and Chi-squared test were performed between groups to analyze patients’ characteristics and determine prognostic factors. A *p*-value of < 0.05 was considered statistically significant.

## 5. Conclusions

In conclusion, based on the neonatal outcome of VRE colonization that occurred after the cervical cerclage, the prognosis could be poor. In comparison, the neonatal outcome in the ESBL-producing bacterial colonization group was not significantly different to that of the no bacterial growth group. However, when early-onset sepsis occurs in neonates after delivery, it can be extremely fatal and can lead to neonatal death. Since antibiotics must be used to control intra-amniotic infection after cervical cerclage, it is necessary to be alert and confirm the occurrence of multidrug-resistant bacteria through cervical culture.

## Figures and Tables

**Table 1:** Basic characteristics of patients who underwent emergency cerclage.

	VRE Colonization (*n* = 6)	ESBL-Producing Bacterial Colonization (*n* = 23)	No Bacterial Growth (*n* = 56)	*p*-Value
Maternal age (years)	31.67 ± 4.50	33.96 ± 3.69	33.91 ± 4.53	0.402
Height (cm)	161.83 ± 2.14	162.30 ± 5.90	161.89 ± 4.41	0.852
Weight before pregnancy (kg)	70.20 ± 13.24	62.00 ± 12.52	59.65 ± 8.36	0.023
Weight at cerclage (kg)	74.57 ± 12.51	66.31 ± 12.25	64.77 ± 8.45	0.047
BMI before pregnancy	26.87 ± 5.34	23.49 ± 4.21	22.78 ± 3.29	0.025
BMI at cerclage	28.55 ± 5.21	25.14 ± 4.17	24.75 ± 3.39	0.057
History of conization	0 (0.0%)	1 (4.35%)	5 (8.93%)	0.603
History of dilation and curettage	1 (16.67%)	5 (21.74%)	10 (17.86%)	0.914

VRE, vancomycin-resistant enterococci; ESBL, extended-spectrum beta-lactamase; BMI, body mass index.

**Table 2:** Clinical/laboratory findings during emergency cerclage.

	VRE Colonization (*n* = 6)	ESBL-Producing Bacterial Colonization (*n* = 23)	No Bacterial Growth (*n* = 56)	*p*-Value
Species identified in cervical culture	*Enterococcus faecium* (*n* = 6)	*E. coli* (*n* = 19), *Klebsiella pneumoniae* (*n* = 4)		
The date the bacteria were identified in the culture after surgery (day)	7 (*n* = 6)	4 (*n* = 12), 7 (*n* = 11)		
GA at cerclage (week)	22.1 ± 1.5	21.6 ± 2.5	21.3 ± 3.0	0.479
CL before cerclage (mm)	6.67 ± 7.34	11.30 ± 6.89	13.01 ± 6.76	0.034
Presence or absence of funneling before cerclage	5 (83.33%)	15 (65.22%)	32 (57.14%)	0.410
Fetal membrane exposed to the vagina before cerclage	3 (50.00%)	4 (17.39%)	4 (7.14%)	0.009
Total CL after cerclage (mm)	35.83 ± 13.45	34.65 ± 6.92	35.59 ± 7.94	0.823
Knot to external os length after cerclage (mm)	13.67 ± 3.08	18.30 ± 4.17	19.00 ± 3.89	0.009

VRE, vancomycin-resistant enterococci; ESBL, extended-spectrum beta-lactamase; GA, gestational age; CL, cervical length.

**Table 3:** Neonatal outcomes in patients who underwent emergency cerclage.

	VRE Colonization (*n* = 6)	ESBL-Producing Bacterial Colonization (*n* = 23)	No Bacterial Growth (*n* = 56)	*p*-Value
Yes or no repeat cerclage	4 (66.67%)	1 (4.35%)	2 (3.57%)	0.000
Duration of cerclage to delivery	70.00 ± 48.65	91.83 ± 35.74	104.86 ± 31.82	0.010
GA at delivery (week)	32.0 ± 7.1	35.0 ± 5.2	36.2 ± 4.2	0.038
Delivery before 34 weeks	3 (50%)	4 (17.4%)	8 (14.3%)	0.093
Delivery before 37 weeks	3 (50%)	9 (39.1%)	17 (30.4%)	0.527
Yes or no cesarean section	4 (66.67%)	8 (34.78%)	39 (69.64%)	0.015
Occurrence of pPROM	2 (33.33%)	3 (13.04%)	7 (12.50%)	0.373
FDIU	0 (0%)	2 (8.70%)	1 (1.8%)	0.283
Birth weight (kg)	1851.67 ± 1096.29	2473.64 ± 878.62	2785.18 ± 768.57	0.006
Apgar score				
At 1 min	5.83 ± 2.40	7.23 ± 1.93	7.16 ± 1.90	0.289
At 5 min	7.83 ± 1.33	8.32 ± 2.01	8.41 ± 1.78	0.513
NICU admission	3 (50%)	6 (28.6%)	8 (14.5%)	0.075
Neonatal death	2 (33.3%)	1 (4.8%)	3 (5.5%)	0.039
Early-onset sepsis in neonate	2 (33.3%)	1 (4.8%)	0 (0%)	0.000

VRE, vancomycin-resistant enterococci; ESBL, extended-spectrum beta-lactamase; GA, gestational age; pPROM, preterm premature rupture of membranes; FDIU, fetal death in utero; NICU, neonatal intensive care unit.

## Data Availability

Data sharing is not applicable to this article.
